# Feasibility of Longitudinal ctDNA Assessment in Patients with Uterine and Extra-Uterine Leiomyosarcoma

**DOI:** 10.3390/cancers15010157

**Published:** 2022-12-27

**Authors:** Maggie Zhou, Nam Bui, Richa Rathore, Sumedha Sudhaman, Giby V. George, Allyson K. Malashevich, Meenakshi Malhotra, Minetta C. Liu, Alexey Aleshin, Kristen N. Ganjoo

**Affiliations:** 1 Sarcoma Program, Stanford Health Care, Palo Alto, CA 94304, USA; 2 Natera Inc., 13011A McCallen Pass, Austin, TX 78753, USA

**Keywords:** leiomyosarcoma, circulating tumor DNA, soft tissue sarcomas

## Abstract

**Simple Summary:**

Leiomyosarcomas are the most common soft tissue sarcomas (STS) subtype characterized by increased aggressiveness and early relapse. Current surveillance includes physical exam and imaging which may be inconclusive, confounding and pose radiation risk with repeat imaging. Given the disease severity and aggressiveness, non-invasive biomarkers are needed to monitor disease progression and treatment response to facilitate prompt clinical decision-making. Circulating tumor DNA (ctDNA) is a minimally invasive blood-based biomarker that has shown to be prognostic of disease outcomes in patients with solid tumors. Herein, we investigated the potential utility of ctDNA for longitudinal monitoring of patients with LMS. Our data suggests that longitudinal ctDNA surveillance may be useful for monitoring treatment response in patients with LMS.

**Abstract:**

**Background**: Leiomyosarcomas (LMS) are aggressive malignancies with a propensity for early relapse. Current surveillance modalities include physical exam and imaging; however, radiological response to therapy may only manifest after 4–6 cycles of treatment. Herein, we evaluated the feasibility of longitudinal circulating tumor DNA (ctDNA) assessment in LMS patients to identify disease progression. **Methods**: We performed a retrospective review of patients with LMS who underwent treatment at Stanford Cancer Center between September 2019 and May 2022. ctDNA detection was performed using a personalized, tumor-informed ctDNA assay. Genomic analysis was conducted to characterize tumor mutation burden (TMB) and known driver mutations. **Results**: A total of 148 plasma samples were obtained from 34 patients with uterine (N = 21) and extrauterine (N = 13) LMS (median follow-up: 67.2 (19–346.3) weeks] and analyzed for ctDNA presence. Nineteen patients had metastatic disease. The most frequently mutated driver genes across sub-cohorts were *TP53*, *RB1*, and *PTEN*. Patients were stratified into four sub-cohorts (A-D) based on ctDNA kinetics. ctDNA levels tracked longitudinally with progression of disease and response to therapy. **Conclusion**: Our results indicate that while undetectable ctDNA may suggest a lower likelihood of relapse, ctDNA positivity may indicate progressive disease, enabling closer monitoring of patients for early clinical intervention.

## 1. Introduction

Soft tissue sarcomas (STS) are a group of heterogeneous mesenchymal tumors that account for less than 1% of cancers in the United States [1]. Despite their rarity, the majority of STS are malignant, often recurring locally or distally with a short median time to recurrence within the first 3 years [2]. At the molecular level, sarcomas are genetically diverse, characterized by unique and often complex somatic mutations, commonly including cell cycle genes such as *MDM2*, *TP53*, *RB1*, and *CDK4* [3]. Leiomyosarcoma (LMS), a malignant smooth muscle neoplasm, is one of the most common STS subtypes, accounting for 10% to 20% of all newly diagnosed sarcomas [4,5]. It originates either directly from smooth muscle cells or from precursor mesenchymal stem cells that ultimately differentiate into smooth muscle cells [6]. Although most cases are sporadic, risk factors for LMS include hereditary retinoblastoma [7]. Li-Fraumeni syndrome [8], and radiation exposure [9,10]. LMS is often characterized by the loss of tumor suppressor genes such as *RB1*, *TP53*, or *PTEN* and/or multiple copy number alterations [11]. LMS frequently has an aggressive clinical course, and patients are at high risk for relapse even after initial complete surgical resection, with up to 80% of relapses occurring within the first 2 years of follow-up [12]. Uterine leiomyosarcoma (uLMS), although rarer arises in the smooth muscle layer of the uterus. It has an annual incidence of 0.35–0.64 per 100,000 women in the U.S. [13], and an even more aggressive course with an overall survival of 57% for stage I disease and 16% for stage IV disease [14]. 

The current treatment regimen for localized LMS involves a combination of surgical resection and radiotherapy (RT) to improve local control and functionality [15]. Although considered incurable, inoperable/advanced/metastatic disease may be treated palliatively with various systemic therapies [15]. Similarly, localized uLMS is treated with total hysterectomy (TAH) with/without bilateral salpingo-oophorectomy (BSO), followed by adjuvant therapy in the form of cytotoxic therapy, immunotherapy, hormone therapy, and/or RT depending on disease stage [14,15]. Physical exam and imaging remain the standard for monitoring response to treatment and detecting relapse in sarcomas [15]; however, imaging may prove inconclusive and confound further work-up given that fibrosis secondary to treatment effect may mimic molecular residual disease (MRD). Additionally, objective responses to therapy assessed by imaging may only manifest after 4 to 6 cycles of treatment [16]. This compounded with the absence of prognostic biomarkers makes it challenging for clinicians to decide the duration of treatment before moving on to experimental agents or other regimes [16]. Thus, given this unmet need and the severity of disease and impact on quality of life, biomarkers are needed to monitor disease progression and assess treatment response in order to enhance clinical decision-making.

Circulating tumor DNA (ctDNA) has emerged as a minimally invasive tumor biomarker that can detect residual disease at the molecular level, obviating the need for repeat imaging and risk of radiation exposure. ctDNA analysis has been incorporated into the disease prognostication, treatment stratification and monitoring of therapeutic response across multiple solid malignancies including breast, colon, lung, and bladder cancers [17,18,19,20,21,22,23]. Additionally, longitudinal monitoring of ctDNA dynamics hold promise in identifying disease progression ahead of radiologic assessment in various malignancies [24]. Despite the growing clinical implications of ctDNA, little is known about its utility as a tumor biomarker in STS given the rarity and genomic heterogeneity of sarcomas. Although STS generally have complex karyotypes and low tumor fraction, preliminary research suggests that ctDNA analysis may be useful in certain subtypes of sarcoma, such as LMS and uLMS.

Here, we present a case series of 34 patients with LMS, in which ctDNA analysis proved useful to monitor surveillance and assess disease progression. Using personalized and tumor-informed ctDNA testing, we also highlight four patient cases that demonstrate concordance between ctDNA results and imaging. Finally, although the identification of driver mutations in known genes associated with LMS and uLMS is difficult given the complex genotypes, we describe the tumor mutation burden (TMB) and pathogenic or likely pathogenic driver mutations observed within the various sub-groups of our cohort based on serial ctDNA detection patterns. Given that TMB status and select mutations have been found to be predictive of therapeutic response, we propose that surveying the molecular landscape of LMS and uLMS may improve survival outcomes [25,26]. 

## 2. Materials and Methods

### 2.1. Patient Population

We performed a retrospective analysis of patients (N = 34) with LMS who underwent treatment at Stanford Cancer Center between September 2019 and May 2022. All patients were >18 years at the time of initial diagnosis. This study was approved by the Stanford Institutional Review Board (Stanford Protocol ID: 34465) and conducted in accordance with the principles of the Declaration of Helsinki and ICH guidelines for Good Clinical Practices. 

### 2.2. Specimen Collection and Processing

Specifically, tumor DNA was extracted from formalin-fixed and paraffin-embedded (FFPE) tissue from surgically resected primary tumor for all patients. A single blood sample was collected in a 6 mL EDTA test tube for germline DNA analysis. Whole exome sequencing (WES) was conducted on both samples. Subsequent blood samples for ctDNA analyses were collected in two, 10 mL Streck tubes at various time points when patients presented in the clinic throughout their treatment/observation regimen.

### 2.3. Personalized, Tumor-Informed ctDNA Detection

ctDNA detection was performed using a personalized, tumor-informed multiplex (m) PCR-based, next-generation sequencing ctDNA assay as described previously (Signatera^TM^, bespoke mPCR, NGS assay) [20]. Briefly, up to 16 tumor-specific, clonal, somatic, single nucleotide variants (SNVs) were tracked in plasma, based on upfront WES of tumor tissue and matched normal blood. Multiplex PCR primers were created based on the selected set of variants. Cell-free DNA (cfDNA) was extracted following a CLIA-validated standard operating procedure at Natera, Inc. Following cfDNA extraction, universal libraries were created by end repair, A-tailing, and ligation with custom adapters. The libraries were amplified using multiplex PCR, barcoded, pooled, and sequenced on the HiSeq 2500 system, Illumina, Inc. NGS platform. Plasma samples with ≥2 SNVs detected were considered ctDNA-positive, and ctDNA concentration was reported in mean tumor molecules (MTM)/mL of plasma. Serial time points were collected to enable correlations between ctDNA levels and response to treatment.

### 2.4. Genomic Analysis to Assess TMB and Identify Driver Mutations

To evaluate TMB based on somatic SNVs per megabase (Mb), whole exome variant call format (VCF) files were analyzed as previously described [27]. These data were also used to identify mutations in known driver genes associated with LMS [28,29,30,31,32,33,34]. 

## 3. Results

### Patient Cohort

Plasma samples (N = 148) were collected from a total of 34 patients with LMS (median age at diagnosis, 52 (range: 34–82) years; 91% female) and were included in the ctDNA analysis. A total of 21 patients had uLMS, while 13 had extrauterine LMS. Median follow-up for the entire patient cohort was 67.2 weeks (range 19–346.3 weeks). Detailed patient characteristics are presented in Table S1. Of the 34 patients analyzed, 19 had metastatic disease. Of these, 2/19 patients (#10 and #11) were metastatic at diagnosis. Patient #10 had not yet started therapy at the time of first ctDNA collection, while patient #11 was pretreated at the time of first ctDNA collection. Two patients (#17 and #34) were newly recurrent and thus untreated at the time of initial ctDNA collection. Patient #21 was known to be metastatic and declined systemic therapy and so was untreated. The remaining 14 patients were diagnosed initially with localized disease, underwent primary surgical resection followed by debulking procedures, and were pretreated at the time of initial ctDNA collection.

For the purpose of data analysis and interpretation, patients were stratified into four sub-cohorts based on ctDNA pattern. Sub-cohort A consisted of patients who tested negative at all time points (N = 12). Sub-cohort B consisted of patients who were initially ctDNA-negative and later became positive (N = 4). Patients in sub-cohort C remained ctDNA-positive throughout follow-up (N = 11). Finally, sub-cohort D consisted of ctDNA-positive patients who became and remained negative or later turned positive (N = 7).

**Figure 1:**
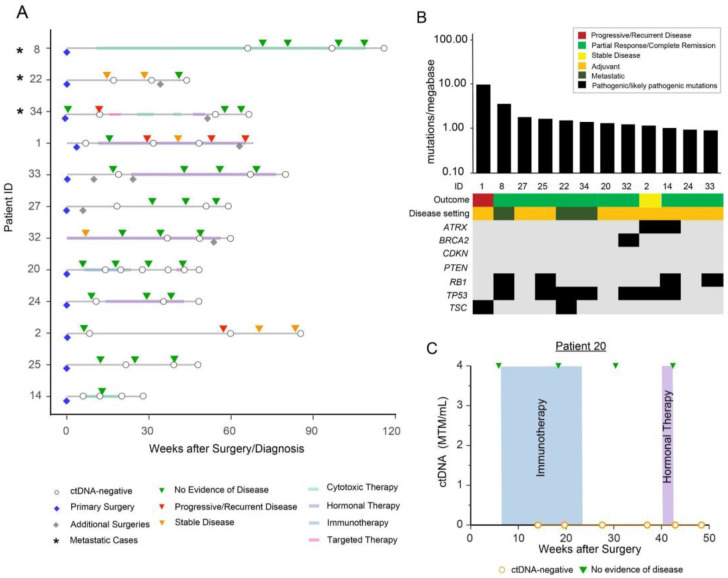
(**A**) Overview plot depicting the complete clinical course of all patients within sub-cohort A (patients who tested negative for ctDNA at all time points), including results of longitudinal ctDNA analysis. Patients with metastatic disease are marked with an asterisk “*” symbol. Blue diamond reflects initial/primary surgery performed to build the personalized ctDNA assay, all other additional surgeries are represented with grey diamonds. (**B**) Distribution of pathogenic and likely pathogenic driver mutations across all 12 patients in sub-cohort A. (**C**) Clinical course for patient #20 depicting immunotherapy and hormonal therapy, with radiologic surveillance and ctDNA levels over time. The debulking procedure prior to the initiation of immunotherapy was used to build a personalized ctDNA assay.

Patients in sub-cohort A tested negative for ctDNA at all time points (N = 12) (Figure 1A). Of the 12 patients, 83.3% (10/12) did not demonstrate clinical relapse/progression by imaging, while 16.7% (2/12) relapsed. TMB status was calculated for all twelve patients within this sub-cohort and a median TMB of 1.39 mutations per Mb (range 0.9–10.05 mutations per Mb) was observed (Figure 1B). Of the twelve patients, 75% (9/12) of patients were observed to possess pathogenic or likely pathogenic mutations in known sarcoma driver genes. WES results revealed the most frequently mutated genes to be *TP53* (58.3%), *RB1* (33.3%), *ATRX* (16.7%), *TSC* (16.7%), and *BRCA2* (8.3%). Interestingly, *TSC* mutations were only observed in this sub-cohort. 

Here, we describe the clinical course of patient #20, a 55-year-old female diagnosed with high-grade LMS, who underwent a TAH/BSO. Postoperatively, she received six cycles of adjuvant chemotherapy in the form of gemcitabine/docetaxel. PET-CT three years after diagnosis revealed a pelvic mass suspicious for recurrence. Laparoscopic resection and pathological examination confirmed recurrent high-grade uLMS. Post-surgery, she received five cycles of adjuvant doxorubicin and subsequently showed progressive disease. She was switched to pazopanib and later received seven cycles of dacarbazine and gemcitabine. She later underwent a debulking surgery, and tissue from this procedure was utilized to build a personalized ctDNA assay (Figure 1C).

Following surgery, the patient was started on ipilimumab/nivolumab (six cycles). She remained ctDNA-negative throughout the duration of treatment. Hormonal therapy with anastrozole was then initiated but discontinued after two weeks due to unwanted side effects. Scans during and after ipilimumab/nivolumab treatment and hormonal therapy demonstrated no evidence of disease, correlating with negative ctDNA results.

**Figure 2:**
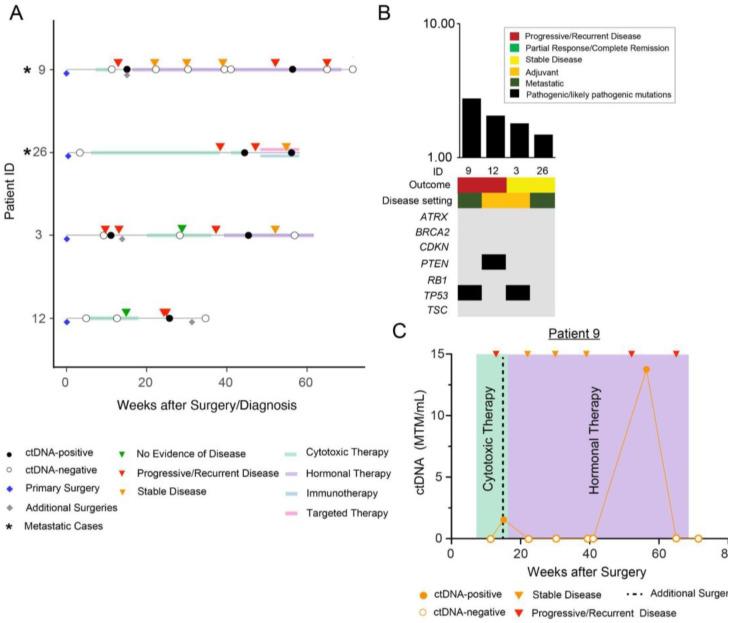
(**A**) Overview plot depicting the complete clinical course of all patients within sub-cohort B (initially ctDNA-negative but later became positive), including results of longitudinal ctDNA analysis. Patients with metastatic disease are marked with an asterisk “*” symbol. Blue diamond reflects initial/primary surgery performed to build the personalized ctDNA assay, all other additional surgeries are represented with grey diamonds. (**B**) Distribution of pathogenic and likely pathogenic driver mutations across the 4 patients in sub-cohort B. (**C**) Clinical course for patient #9 who underwent an initial TAH/BSO for an ER/PR+, grade 2 uLMS, followed by cytotoxic therapy, and several metastatic resections (not depicted). Tissue from her sixth debulking procedure (denoted as additional surgery) was used to develop a personalized ctDNA assay. She subsequently received additional chemotherapy and RT (not shown) and hormonal therapy (noted in purple).

Patients in sub-cohort B were initially ctDNA-negative but later became positive (N = 4) (Figure 2A). After calculating TMB status for patients within this sub-cohort, a median TMB of 1.92 mutations/Mb (range 1.48–2.76 mutations per Mb) was observed (Figure 2B). Three of four patients within this sub-cohort were observed to have pathogenic or likely pathogenic mutations in driver genes including *TP53* (50%) and *PTEN* (25%).

In this sub-cohort we describe the clinical course of patient #9, a 62-year-old female with metastatic uLMS. The patient underwent a TAH/BSO for a large abdominal mass identified on MRI, and pathological diagnosis revealed an estrogen receptor/progesterone receptor-positive (ER/PR+), grade 2 uLMS with negative margins. Post-surgery, she received cytotoxic therapy with six cycles of gemcitabine and docetaxel and her disease remained stable by imaging. Two years after initial diagnosis, CT chest revealed nodules suspicious for metastatic recurrence. A wedge resection revealed a spindle cell neoplasm consistent with metastatic leiomyosarcoma. In a clinical trial with aldoxorubicin versus dacarbazine, she was randomized to dacarbazine for three cycles.

Following multiple resections and lines of systemic therapy, she underwent a sixth surgery consisting of tumor debulking with hyperthermic intraperitoneal chemotherapy (HIPEC) and abdominal wall reconstruction. Tissue from this procedure was used to design a personalized ctDNA assay (Figure 2C). Following surgery, she received three doses of eribulin, after which ctDNA testing demonstrated no detectable ctDNA. The patient then underwent another resection of her abdominal wall tumors.

After the resection, ctDNA levels increased to 1.54 MTM/mL. At this time, the patient received three weeks of palbociclib, RT for liver metastases, and hormonal therapy in the form of letrozole followed by fulvestrant. Subsequently, ctDNA levels again became negative, and then became positive (13.76 MTM/mL) 10 months after resection, corresponding to progressive metastatic disease on imaging. At her most recent clinical follow-up 10 years after diagnosis, she remains alive with active metastatic disease and is being maintained on palbociclib and gemcitabine.

**Figure 3:**
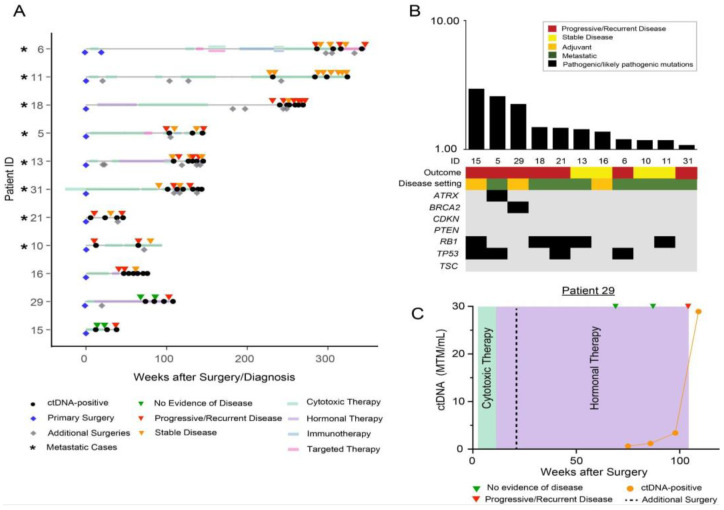
(**A**) Overview plot depicting the complete clinical course of all patients within sub-cohort C, including the results of longitudinal ctDNA analysis. Patients with metastatic disease are marked with an asterisk “*” symbol. Blue diamond reflects initial/primary surgery performed to build the personalized ctDNA assay, all other additional surgeries are represented with grey diamonds. (**B**) Distribution of pathogenic and likely pathogenic driver mutations across all 11 patients in sub-cohort C. (**C**) Clinical course for patient #29 who underwent an initial robotic supracervical hysterectomy and bilateral salpingectomy for a weakly ER/PR-positive uLMS and subsequent robotic assisted trachelectomy, infracolic omentectomy with bilateral pelvic lymph node dissection (not shown). A sample from a CT-guided biopsy the following year was used to build a personalized ctDNA assay. She later received cytotoxic therapy, followed by a metastatic resection, and hormonal therapy.

Sub-cohort C consisted of patients who tested positive at all time points (N = 11) (Figure 3A). All 11 patients (100%) had ctDNA positivity at the last time point which was concordant with evidence of disease on imaging. TMB analysis revealed a median TMB of 1.43 mutations/Mb (range 1.08–2.94 mutations per Mb) in this sub-cohort (Figure 3B). Nine of twelve (75%) of patients were observed to have pathogenic or likely pathogenic mutations in driver genes. The most prevalent mutations were observed to be in the *RB1* (45.5%), *TP53* (36.4%), *BRCA2* (9%), and *ATRX* (9%) genes.

In this sub-cohort we describe the clinical course of patient #29, a 45-year-old female who underwent a robotic supracervical hysterectomy and bilateral salpingectomy for a pathology-confirmed 7 cm uLMS within the myometrium with weak ER/PR-positivity (stage pT1 bNx). Two months later, she underwent a robotic assisted trachelectomy and infracolic omentectomy with bilateral pelvic lymph node dissection. CT-guided biopsy of a suspicious retroperitoneal mass the following year revealed recurrent uLMS. This sample was utilized to build a personalized ctDNA assay (Figure 3C). She was started on doxorubicin with dexrazoxane and subsequently underwent a bilateral oophorectomy with mesenteric mass resection that proved positive for metastatic uLMS. Hormonal therapy was initiated with anastrozole. Notably, ctDNA-positivity identified disease recurrence ahead of radiographic findings in this patient with a lead time of 29.1 weeks. More recently, she underwent an exploratory laparotomy with extensive tumor debulking and HIPEC. At her latest follow-up appointment, she remains alive and is receiving adjuvant dacarbazine (4 cycles) as part of an ongoing clinical trial.

**Figure 4:**
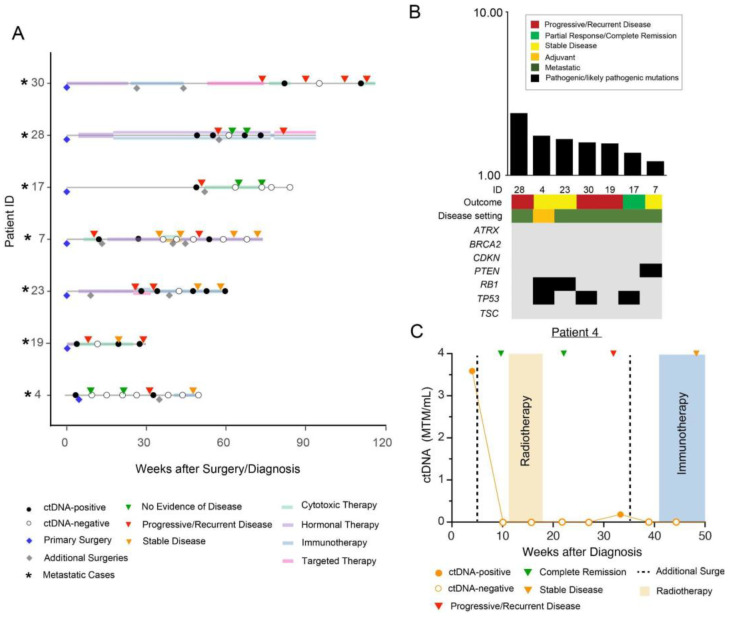
(**A**) Overview plot depicting the complete clinical course of all patients within sub-cohort D, including the results of longitudinal ctDNA analysis. Patients with metastatic disease are marked with an asterisk “*” symbol. Blue diamond reflects initial/primary surgery performed to build the personalized ctDNA assay, all other additional surgeries are represented with grey diamonds. (**A**) Distribution of pathogenic and likely pathogenic driver mutations across all 7 patients in sub-cohort D. (**C**) Clinical course for patient #4 depicting initial surgery for high-grade LMS. A biopsy sample prior to surgery was used to design a personalized ctDNA assay. The patient received adjuvant RT post-surgery followed by a subsequent metastatic resection and immunotherapy.

Finally, sub-cohort D consisted of ctDNA-positive patients who became and remained negative or later turned positive (N = 7) (Figure 4A). The median TMB was observed to be 1.59 mutations/Mb (range: 1.2–2.39 mutations per Mb) in this sub-cohort (Figure 4B). Of the seven patients in sub-cohort D, five (71.4%) showed pathogenic or likely pathogenic mutations in driver genes, including *TP53* (42.9%), *RB1* (28.6%), and *PTEN* (14.3%).

Figure 4C displays the clinical course of patient #4, a 64-year-old male with neurofibromatosis (NF) type 1 who presented with a rapidly enlarging soft tissue thigh mass, biopsy-proven to be high-grade LMS. This sample was used to design a personalized ctDNA assay. He was ctDNA-positive prior to surgery with ctDNA levels of 3.59 MTM/mL. After surgical resection of the mass, he received adjuvant radiation (5940 cGy in 28 fractions) and remained disease-free by both ctDNA and imaging until week 34, at which time surveillance imaging revealed an enlarging 1 cm left lower lobe lung nodule, which was concerning for recurrence. At this point, he also became ctDNA-positive (0.18 MTM/mL). Left lower lobe wedge resection confirmed metastatic LMS, and the patient became ctDNA negative after this resection. At his most recent clinical follow-up, over a year after primary surgical resection, the patient remains alive and disease-free and is being maintained on pembrolizumab.

## 4. Discussion

In this retrospective study, we investigated the potential prognostic utility of ctDNA monitoring in patients with LMS and uLMS. Using four patient cases, we demonstrate that ctDNA-positivity generally corresponds to radiographic confirmation of disease progression, suggesting that the frequency of imaging may be reduced to avoid unnecessary radiation exposure.

We observed that ctDNA levels tracked longitudinally with progression or stabilization of disease and response to therapy. In fact, in select patients, such as patient #29, ctDNA-positivity preceded radiologic confirmation of disease recurrence, enabling a lead-time for clinical intervention. Thus, in the post-surgical setting for LMS and uLMS, ctDNA testing for MRD may be a valid tumor biomarker for predicting disease recurrence and facilitating adjuvant therapy selection and decision-making. ctDNA-positivity in the absence of radiographic findings of disease may heighten clinical suspicion for recurrence, and in the appropriate clinical setting may encourage the initiation or intensification of adjuvant therapy.

Although research regarding the utility of ctDNA as a tumor biomarker in LMS is limited, our results demonstrating ctDNA dynamics to track with disease burden when monitored longitudinally is consistent with published data. In a retrospective cohort of patients with LMS, Hemming and colleagues demonstrated that ctDNA was accurately identified in 69% (11/16) of patients with disease progression and high disease burden (>5 cm), while ctDNA was unidentifiable in patients with stable disease or low tumor burden [11]. More recently, this research was expanded upon to determine correlations between ctDNA levels, treatment response and outcome in patients with advanced LMS. Using plasma samples obtained from 230 patients enrolled in the SARC021 trial, Madanat-Harjuoja et al. found that patients with ctDNA-positivity had a significantly lower 1-year survival compared to ctDNA-negative patients (HR: 1.77, 95% CI: 1–3.14, *p* = 0.049) [16]. ctDNA-positive patients also had a lower 1-year progression-free survival compared to ctDNA-negative patients, albeit statistically insignificant (HR: 1.2, 95% CI: 0.6–2.37, *p* = 0.61) [16]. This preliminary research suggests that monitoring ctDNA levels in LMS has important implications in the care of patients with metastatic and progressive LMS and therefore may enable reliable measurement of disease burden and response to therapy.

Our genomics analysis revealed that of the four subgroups within our cohort, the highest variability in the range of TMB was seen within sub-cohort A, which had serially negative ctDNA. This was also the subgroup that had the best clinical outcome relative to the other subgroups. This information may prove clinically relevant, as high TMB is an established biomarker of improved immune checkpoint blockade (ICB) response [26]. Similar to published literature, our findings demonstrate the most frequently mutated driver genes across sub-cohorts to be *TP53, RB1, and PTEN* [35]. The frequency of mutations in known driver genes was again noted to be highest within sub-cohort A. Although no targeted therapy options currently exist for these mutations, their identification provides insight into the molecular landscape of LMS and the possibilities for treatment. Of note, poly ADP ribose polymerase inhibitors (PARPi) may prove beneficial in homologous recombination (HR) proficient but *PTEN* and/or *ATRX* deficient uLMS, stressing the need for genetic testing in uterine, as well as extrauterine, LMS [25]. While no significant association was seen between serial ctDNA patterns and the presence of driver mutations in our cohort, additional analyses using a larger cohort are needed to understand the implications of these molecular findings.

LMS has a high unmet need given its extremely poor prognosis, especially in the metastatic setting [36]. Early detection of relapse is critical to improving patient outcomes. Data remain limited regarding patient selection and the timing of adjuvant chemotherapy following primary surgical resection. To date, no large, randomized studies have been conducted to guide clinical management, largely due to the rarity of the disease which limits patient recruitment. ctDNA testing may be especially useful in this setting to both identify patients most likely to benefit from therapy and to decide whether to extend or hold-off on further therapy. The collection of presurgical blood draws would allow the quantification of baseline ctDNA detection rates and would inform the efficacy of neoadjuvant treatment and post- surgical decision making. Additionally, undetectable ctDNA levels may suggest a lower likelihood of relapse and close monitoring may enable identification of relapse before radiographic recurrence. Our case series study possesses several limitations. Our cohort of patients was small, and ctDNA associations with disease burden and response to therapy are descriptive. Given the retrospective nature of this investigation, we were unable to control for confounders that may have affected patient selection for ctDNA testing. Furthermore, overall ctDNA detection may have been impacted by non-shedding tumors, the timing of ctDNA testing, indolent disease, and overall tumor burden.

## 5. Conclusions

In summary, we highlight the potential role for ctDNA in patients with LMS which may enable personalized disease management in future clinical trials. If validated, longitudinal ctDNA monitoring in the adjuvant setting for the purpose of informing treatment decisions and prediction of disease recurrence during the surveillance period may allow timely decision-making and improve survival outcomes in LMS.

## Data Availability

All data generated during this study are included in this article. Further enquiries can be directed to the corresponding author.

## Supplementary Materials

The following supporting information can be downloaded at: https://www.mdpi.com/article/10.3390/cancers15010157/s1, Table S1: Patient Characteristics.
