# Supplementary material for: Feasibility of Longitudinal ctDNA Assessment in Patients with Uterine and Extra-Uterine Leiomyosarcoma
Source: Cancers (Basel). 2022 Dec 27;15(1):157. doi: 10.3390/cancers15010157 (PMC9818540; doi:10.3390/cancers15010157)
Supplement: Supplementary file 1 [file cancers-15-00157-s001.zip › cancers-2112150-supplementary.pdf]

Feasibility of longitudinal ctDNA assessment in patients with uterine and extra-uterine leiomyosarcoma

Maggie Zhou[1], Nam Bui[1], Richa Rathore[2], Sumedha Sudhaman[2], Giby V. George[2], Allyson K. Malashevich[2], Meenakshi Malhotra[2], Minetta C. Liu[2], Alexey Aleshin[2] and Kristen N. Ganjoo[1]*

Table S1. Patient Characteristics

Patient ID	Age at Diagnosis (years)	Sex (F/M)	Disease Setting (Adjuvant/Metastatic)	Disease Location (Intra/Extra-uterine)	Survival Status (Alive/Deceased)	Duration of Follow-up (weeks)	Disease Recurrence/Progression (PD/recurrence, PR/CR, SD)	ctDNA at Last Timepoint	Therapy group (Hormonal, Cytotoxic, Immunotherapy, Targeted)	Mutational Status
1	55.8	F	Adjuvant	Extra-uterine	Alive	64.4	PD/recurrence	Negative	Hormonal	TSC
2	55.6	F	Adjuvant	Intra-uterine	Alive	85.9	SD	Negative	N/A	ATRX, TP53
3	61.4	F	Adjuvant	Intra-uterine	Alive	61.1	SD	Negative	Cytotoxic, hormonal	TP53
4	64.0	M	Adjuvant	Extra-uterine	Alive	45.0	SD	Negative	Immunotherapy	-
5	39.9	F	Metastatic	Extra-uterine	Alive	149.0	PD/recurrence	Positive	Cytotoxic, targeted, immunotherapy	ATRX, TP53
6	51.3	M	Metastatic	Extra-uterine	Alive	347.7	PD/recurrence	Positive	Cytotoxic, targeted, immunotherapy	TP53

7	52.2	F	Metastatic	Intra-uterine	Alive	73.9	SD	Negative	Cytotoxic, hormonal	-
8	56.1	F	Metastatic	Intra-uterine	Alive	108.9	PR/CR	Negative	Cytotoxic	RB1, TP53
9	51.8	F	Metastatic	Intra-uterine	Alive	68.6	PD/recurrence	Negative	Cytotoxic, hormonal	TP53
10	50.1	F	Metastatic	Extra-uterine	Alive	94.3	SD	Positive	Cytotoxic	-
11	58.6	M	Metastatic	Extra-uterine	Alive	323.6	SD	Positive	Cytotoxic	RB1
12	48.2	F	Adjuvant	Intra-uterine	Alive	33.7	PD/recurrence	Negative	Cytotoxic	PTEN
13	53.3	F	Metastatic	Intra-uterine	Alive	142.9	SD	Positive	Cytotoxic, hormonal, immunotherapy, targeted	RB1
14	60.2	F	Adjuvant	Intra-uterine	Alive	19.0	PR/CR	Negative	Cytotoxic	ATRX, RB1, TP53
15	50.3	F	Adjuvant	Intra-uterine	Alive	37.1	PD/recurrence	Positive	Cytotoxic, targeted	RB1, TP53
16	35.9	F	Adjuvant	Intra-uterine	Alive	62.9	SD	Positive	Cytotoxic, hormonal	-
17	82.2	F	Metastatic	Intra-uterine	Alive	78.1	PR/CR	Negative	Cytotoxic	-
18	63.4	F	Metastatic	Intra-uterine	Alive	268.0	PD/recurrence	Positive	Hormonal, cytotoxic, targeted	RB1

19	68.8	F	Metastatic	Intra-uterine	Alive	118.6	PD/recurrence	Positive	Hormonal, cytotoxic, targeted, immunotherapy	-
20	48.6	F	Adjuvant	Extra-uterine	Alive	42.9	PR/CR	Negative	Immunotherapy, hormonal	-
21	71.7	F	Metastatic	Extra-uterine	Alive	49.9	PD/recurrence	Positive	N/A	RB1, TP53
22	44.3	F	Metastatic	Intra-uterine	Alive	41.1	PR/CR	Negative	N/A	TP53, TSC
23	66.9	F	Metastatic	Extra-uterine	Alive	58.4	SD	Positive	Hormonal, targeted, immunotherapy	-
24	48.7	F	Adjuvant	Intra-uterine	Alive	42.7	PR/CR	Negative	Hormonal	-
25	65.5	F	Adjuvant	Extra-uterine	Alive	39.3	PR/CR	Negative	N/A	RB1, TP53
26	63.5	F	Metastatic	Intra-uterine	Alive	57.6	SD	Positive	Cytotoxic, Targeted, Immunotherapy	-
27	52.2	F	Adjuvant	Extra-uterine	Alive	59.0	PR/CR	Negative	N/A	-
28	51.8	F	Metastatic	Extra-uterine	Alive	93.9	PD/recurrence	Positive	Immunotherapy, Targeted	-
29	42.1	F	Adjuvant	Intra-uterine	Alive	109.1	PD/recurrence	Positive	Cytotoxic, Hormonal	BRCA2

30	34.2	F	Metastatic	Intra-uterine	Deceased	116.3	PD/recurrence	Positive	Hormonal, Immunotherapy, Targeted, Cytotoxic	-
31	42.1	F	Metastatic	Intra-uterine	Alive	137.4	PD/recurrence	Positive	Cytotoxic, Targeted	-
32	48.4	F	Adjuvant	Extra-uterine	Alive	55.9	PR/CR	Negative	Hormonal	BRCA2, TP53
33	46.9	F	Adjuvant	Intra-uterine	Alive	76.1	PR/CR	Negative	Hormonal	RB1
34	50.7	F	Metastatic	Intra-uterine	Alive	66.4	PR/CR	Negative	Targeted, Cytotoxic, Hormonal	TP53

Abbreviations: F; Female, M; Male, PD; progressive disease, PR; partial response, CR; complete remission, SD; stable disease, N/A; not applicable.
